# The *Drosophila tyramine beta-hydroxylase* gene is required for ethanol tolerance

**DOI:** 10.1038/s41598-026-45082-3

**Published:** 2026-04-11

**Authors:** Manuela Ruppert, Stefanie Hampel, Gerbera Claßen, Claire Fuchs, Thomas Kell, Sravya Paluri, Henrike Scholz

**Affiliations:** 1 Department of Biology, Institute for Zoology, University of Köln, Zülpicher Straße 47b, 50674 Cologne, Germany; 2 Biozentrum, Institute for Genetics and Neurobiology, University of Würzburg, 97074 Würzburg, Germany; 3 UPR School of Medicine, Institute of Neurobiology, San Juan, Puerto Rico 00901 USA; 4 Department of Zoology, University of British Columbia, Vancouver, BC V6T 1Z3 Canada

**Keywords:** Tbh, Locomotion, Alcohol tolerance, Stress sensitivity, Octopamine, *Drosophila melanogaster*, Genetics, Molecular biology, Neuroscience

## Abstract

Tyramine beta-hydroxylase (Tbh) is required for octopamine synthesis. To better understand the function of Tbh in neurotransmitter synthesis in *Drosophila melanogaster*, we analyzed the molecular genetic organization of the *Tbh* gene and found that it encodes multiple transcripts. The transcripts differed in their 5′ untranslated region, and the resulting proteins differed in size. We identified new *Tbh* alleles and generated a new *D. melanogaster Tbh* mutant, *Tbh*^*Del3*^, using FLP/FRT recombination mutagenesis to remove the translational start site that was still present in *Tbh*^*nM18*^ mutants. The *Tbh*^*Del3*^ mutants shared ethanol tolerance and larval locomotion defects with the *Tbh*^*nM18*^ mutants. However, they differed in their cellular stress response. While *Tbh*^*nM18*^ mutants exhibited increased ethanol resistance following heat shock, the *Tbh*^*Del3*^ mutant did not achieve a similar level of resistance. To develop normal levels of ethanol tolerance, Tbh is required in a subset of neurons in the adult brain, which was identified with a newly generated *Tbh*-Gal4 driver. Both *Tbh* overexpression and reduction resulted in reduced tolerance, suggesting that Tbh levels need to be tightly controlled. These results improve our understanding of neurotransmitter synthesis and Tbh-dependent behavior.

**Supplementary Information:**

The online version contains supplementary material available at 10.1038/s41598-026-45082-3.

## Introduction

The octopaminergic neurotransmitter system regulates physiological processes ranging from energy metabolism to behavior. The rate-limiting enzyme in *D. melanogaster* that is required for octopamine synthesis is the Tyramine beta-hydroxylase (Tbh) enzyme, which efficiently hydrolyzes tyramine to octopamine^1,2^. The originally identified Tbh isoform shares approximately 39% identity with mammalian Dopamine beta*-*hydroxylase (Dbh;^1^). In vertebrates, Dbh catalyzes the hydroxylation of dopamine to noradrenaline, a molecule structurally very similar to octopamine^3^. To analyze the function of octopamine in regulating physiological processes in *D. melanogaster*, mutants were generated with P-element excision mutagenesis^1^. One of these mutants, the *Tbh*^*nM18*^ mutant, had no detectable level of octopamine and an approximately tenfold increase in tyramine levels^1^. Initial phenotypic characterization of this mutant revealed that Tbh is required for female fertility, a function to which numerous octopaminergic neurons in the thoracic–abdominal ganglion of the adult female fly contribute^1,4^. This mutant is now intensively used to study the function of Tbh and/or octopamine in the regulation of cellular processes and behavior during development or in the adult nervous system.

Tbh is required to initiate and adjust behaviors but does not appear to be directly involved in the performance of motor behavior. For example, adult *Tbh*^*nM18*^ flies can run as fast as controls can but show reduced initiation of locomotion, such as aggression or flight activity^5–7^. They show a lower probability of extending their proboscis in response to sucrose. However, when they do extend their proboscis, they habituate just as effectively as the control flies do^8^. A strong activator of locomotor activity is ethanol^9–11^. Brief exposure to the odorant ethanol induces an olfactory startle response, measured by increased locomotor activity^10,5^. The startle response is strongly reduced in *Tbh*^*nM18*^ mutants and is due to loss of octopamine function^5^. In contrast to the initial reduction in locomotor activity, prolonged ethanol exposure leads to increased locomotor activity in adult *Tbh*^*nM18*^ mutants^5^. Our previous work revealed that approaching an attractive stimulus, such as ethanol, requires the function of Tbh in a subset of Tbh-positive neurons^12,13^. More specifically, this innate attraction to ethanol—referred to as ethanol preference—depends on Tbh function in neurons of the G3a and/or VM clusters^12^. Compared with control flies, *Tbh*^*nM18*^ mutant flies make random choices, which supports the hypothesis that Tbh is required for the initiation of a selective behavioral response^13^. These results further show that the execution of motor performance is not altered in adult *Tbh*^*nM18*^ mutants but that the initiation to perform the required action is missing.

In addition to a reduced initial behavioral response rate, *Tbh*^*nM18*^ flies fail to adapt their behavior after they have a previous experience. For example, upon receiving a second dose, flies previously exposed to ethanol exhibit increased resistance to its intoxicating effects. This relative increase in resistance is known as ethanol tolerance. *Tbh*^*nM18*^ flies show normal sensitivity to the effect of ethanol on postural control but are unable to increase their resistance to a similar extent as controls and, therefore, develop reduced ethanol tolerance^14^. After multiple exposures to ethanol, they develop ethanol tolerance and eventually reach the same levels as controls do^10^. Accordingly, after chronic, long-term exposure to ethanol, their tolerance does not differ from that of control flies^15^. Although neurons mediating innate ethanol preference have been characterized, the neurons responsible for ethanol tolerance, which depends on prior experience and Tbh function, have yet to be identified.

To further examine the function of Tbh in regulating ethanol tolerance, we first analyzed the molecular nature of the *Tbh* gene. Because we identified additional splice variants of *Tbh* and still detected residual transcripts in the *Tbh*^*nM18*^ mutants, we generated a new *Tbh* allele. We tested the males of these mutants for their ability to develop ethanol tolerance and to respond to cellular stress. In addition, we characterized adult and larval locomotion in response to external stressors and changes in internal conditions. By restoring Tbh expression in *Tbh*^*nM18*^ mutants with a newly generated *Tbh*-promoter-Gal4 driver, we identified the neurons required for tolerance. Our results are significant, as the generation and phenotypic characterization of new *Tbh* mutants, along with the identification of multiple transcripts, provide new insights into the regulation of neurotransmitter synthesis and the regulation of behavior.

## Results

The *Tbh*^*nM18*^ mutant, which lacks the neurotransmitter octopamine, is often used to study the function of Tbh and/or octopamine. This mutant was isolated by P-element excision mutagenesis with the MF372 insertion line used as the starting point. However, the exact molecular change responsible for the mutation has not been described in detail^1^.

### **The*Tbh *gene encodes several transcripts**

**Fig. 1:**
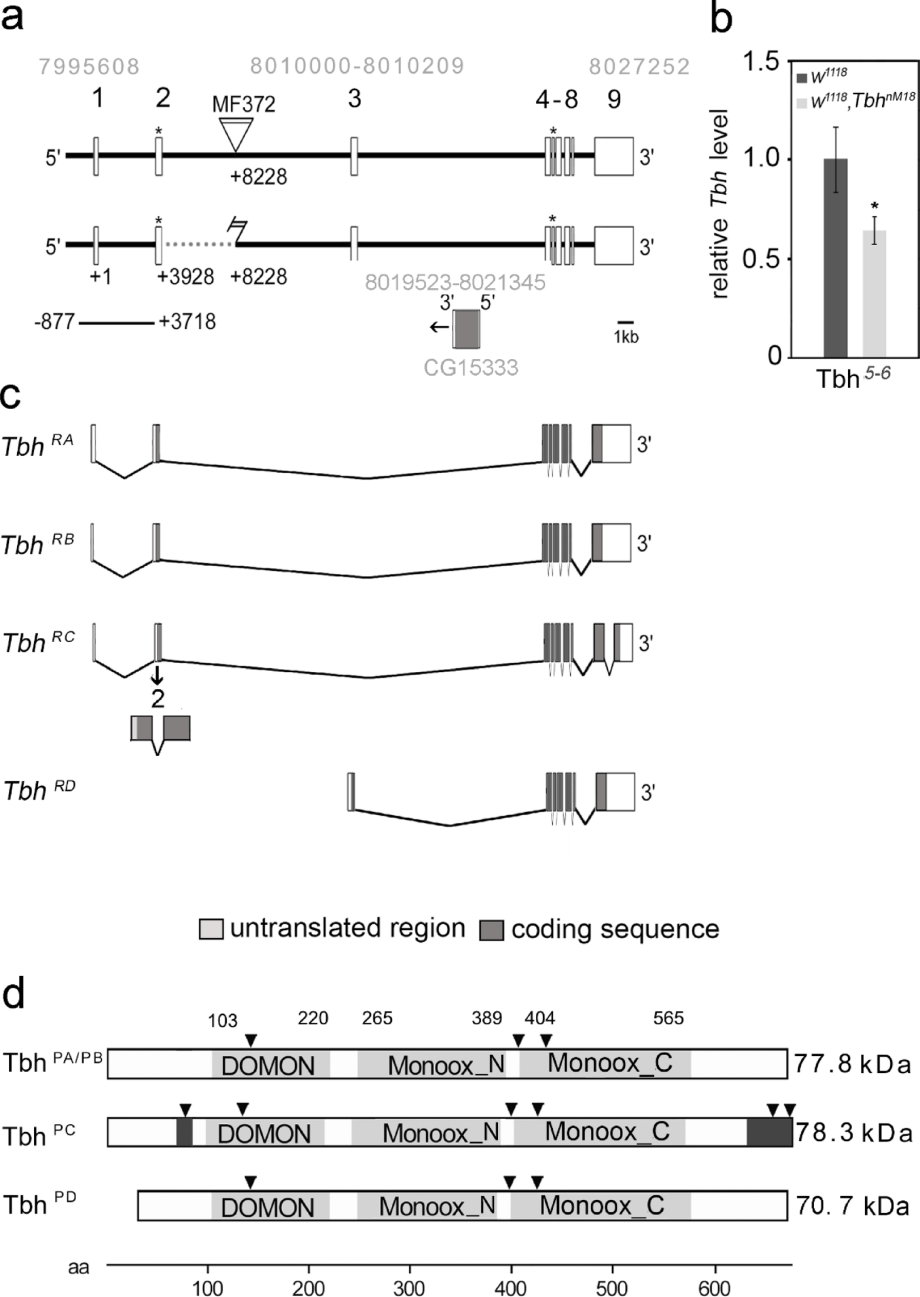
The *Tbh* gene encodes several isoforms. The genomic organization of the Tbh gene spans 31 kb. The triangle indicates the transposable element of the *Tbh*^*MF372*^ insertion, and the light gray numbers indicate the genomic region of the Tbh gene and the position of the newly identified third exon. The dotted line represents the lesion of the mutant *Tbh*^*nM18*^ allele. The deletion includes 32 bp of the second exon but does not include the translational start sites marked with the asterisks. The bar below indicates the genomic region used to generate the 4.6-*Tbh*-Gal4 driver. (**B**) qRT‒PCR analysis of poly (**A**) selected RNA from *Tbh*^*nM18*^ heads with *Tbh* primers specific to the transition between exons 5 and 6 shows *Tbh* transcripts in the mutants (N = 4 independent cDNA samples with three replicates; *RpLPO* gene primers were used as a reference; the transcript levels were different (two sample T test (Welch) with T distribution: (df = 3.5037), P = 0.03857; the error bars represent s.e.m.). (**C**) Four *Tbh* splice variants were identified by RT‒PCR analysis. All transcripts had common sequences for the DOMON domain and the two copper-type II ascorbate-dependent monooxygenase domains. (**D**) Tbh^PA/PB^, Tbh^PC^ and Tbh^PD^ share the DOMON domain and two copper type II ascorbate-dependent monooxygenase domains (light gray boxes). The Tbh^PC^ protein has unique amino acid sequences at the N- and C-termini (dark gray boxes). The putative protein kinase C phosphorylation sites are marked with black triangles.

 First, we determined the precise nature of the molecular lesion. Through sequencing analysis, we found that the *Tbh*^*nM18*^ allele had a deletion of 4269 bp, including 32 bp of the coding sequence of the 3′ end of the second exon and the sequence of the second intron (Fig. 1A). The deletion did not include the translation start site of the *Tbh* gene but resulted in a frame shift of the coding sequence. To analyze the consequences of the deletion at the transcript level, we determined the amount of *Tbh* transcript in the mutants with exon-specific primers for the transition between exons 5 and 6 (*Tbh*^5-^^6^ with quantitative real-time PCR (qRT‒PCR; Fig. 1B). In the *Tbh*^*nM18*^ mutant, the *Tbh*^5-^^6^ transcript sequence was still present. The quantity was significantly reduced by approximately 45%. To determine whether additional *Tbh* transcripts exist that may not be affected by the mutation, we analyzed the sequence of expressed sequence tag (EST) clones of *Tbh*. Furthermore, we compared the *Tbh* transcripts of *Periplaneta americana*^16^ with those of *D melanogaster.* In addition, we analyzed the sequence of the second intron for additional open reading frames. To validate the transcripts, we performed RT‒PCR analysis on cDNA isolated from the heads of control flies. We identified four different transcripts and a novel exon within the second intron of the annotated *Tbh* gene (*Tbh*^*RA*^ to *Tbh*^*RD*^; Fig. 1C). The *Tbh*^*RA*^ and *Tbh*^*RB*^ transcripts differed in the length of the 5’ UTR but not in the protein they encode. In the *Tbh*^*RC*^ transcript, the second exon was alternatively spliced. The fourth 1.5 kb-long *Tbh*^*RD*^ transcript contained the sequence of a newly identified exon located after the second annotated exon. In our RT‒PCR analysis, we could not detect any connections between the new third exon and the first and/or second exon of *Tbh*^*RA/RB*^. This suggests that *Tbh*^*RD*^ uses different sequences other than the 5′UTR and a different translational start site.

The four transcripts encoded three different proteins, as the *Tbh*^*RA*^ and *Tbh*^*RB*^ transcripts encoded the same protein. The Tbh^PA/PB^ and Tbh^PC^ isoforms of 77,7 kDA and 78,3 kDA, respectively, shared the DOMON domain and the N- and C-termini of the copper type II ascorbate-dependent monooxygenase domain (Fig. 1D). The DOMON domain is a 110 to 125 amino acid stretch in the N-terminal region of the dopamine beta-monooxygenase that is found in combination with monooxygenase domains^17^. The Tbh^PC^ isoform differed from Tbh^PA/PB^ in its N- and C-terminal regions, which contained three additional putative protein kinase C phosphorylation sites (Fig. 1D). The Tbh^PD^ protein was approximately 70,7 kDA in size and contained a similar C-terminal region and a truncated N-terminus. In summary, the *Tbh* gene transcribed at least four different transcripts and encoded at least three different isoforms. Furthermore, the *Tbh*^*nM18*^ mutant allele did not appear to be a transcriptional null allele.

### The *Tbh*^*Del3*^ mutant had defects in the cellular stress response

**Fig. 2:**
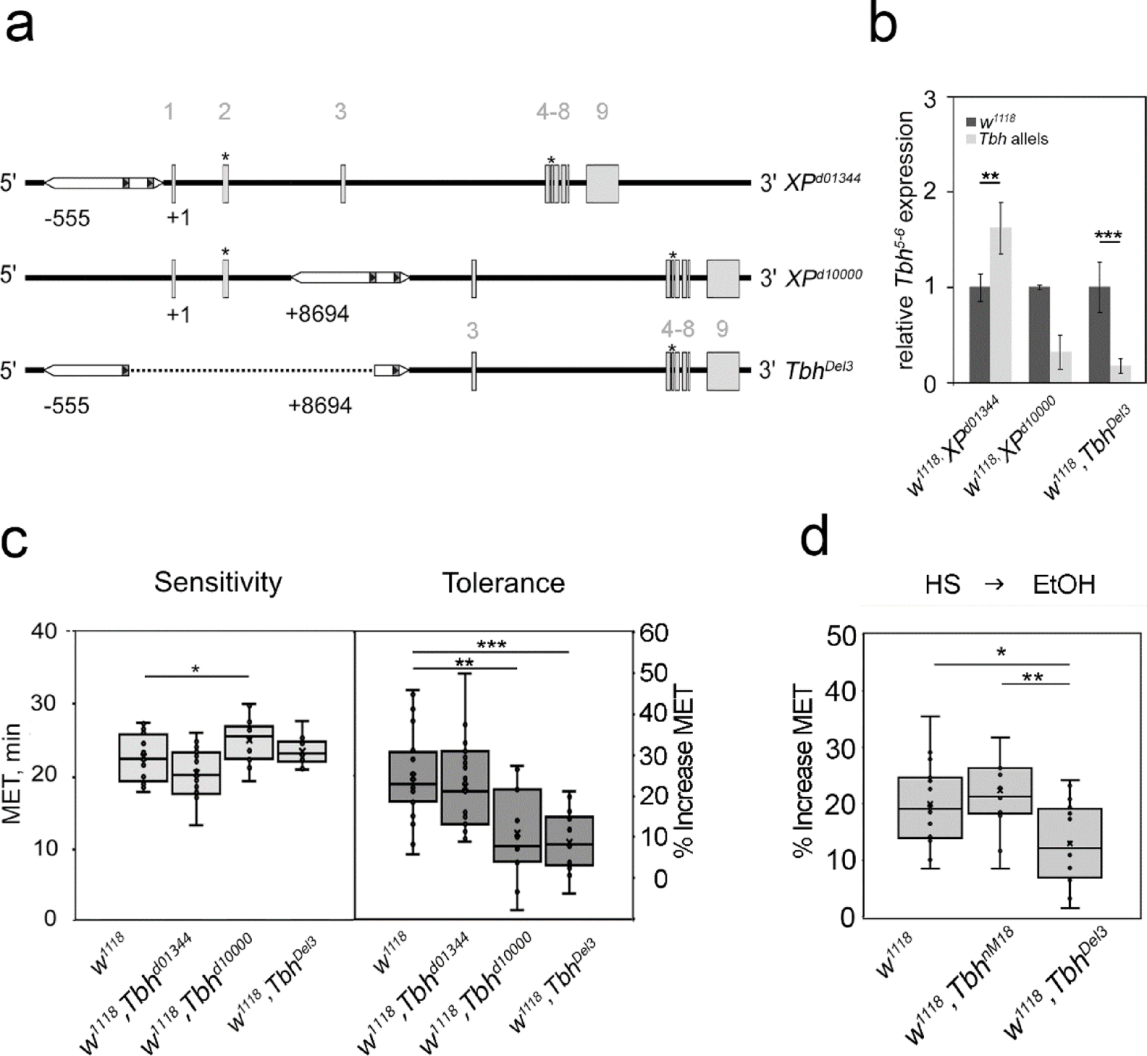
The *Tbh*^*Del3*^ mutant is impaired in the cellular stress response. (**A**) The genomic organization of the *XP*^d01344^ and *XP*^d10000^ lines used to generate the *Tbh*^Del3^ mutant is shown. The dotted line indicates the deletion of the *Tbh*^Del3^ allele generated by FRT/Flip-based recombination. (**B**) qRT‒PCR analysis of *XP*^d01344^, *XP*^d10000^ lines and the *Tbh*^Del3^ mutant revealed significant up- or downregulation of *Tbh* transcripts (N = 3–4 independent cDNA samples with three replicates; *RpLPO* gene primers were used as a reference; two sample T test (Welch): for XPd01344, (df = 10.3126), *P* = 0.005801; for *XP*^d10000^, (df = 11.8928), *P* = 0.3454; and for *Tbh*^Del3^, (df = 8.8554), *P* = 4.776e-7; the error bars represent s.e.m.). (**C**) Compared with the controls, the *XP*^d10000^ mutant was significantly more resistant to ethanol. ANOVA (F (3,68) = 10.1, *P* = 0.000013). The*Tbh*^Del3^ and *TbhXP*^d10000^ mutants developed similarly significantly reduced ethanol tolerances. ANOVA (F (3,68) = 10.1, *P* = 0.000013). (N = 14–23). (**D**) After heat shock, the control and *Tbh*^nM18^ flies were more resistant to ethanol. *Tbh*^Del3^ mutants developed significantly less resistance to ethanol. ANOVA (F (2,44) = 6.69, *P* = 0.002899. (N = 14–17). To calculate heat–ethanol cross-tolerance, the MET of heat-shocked flies was compared to the MET1 of flies of the same genotype without heat shock. Significance was determined with ANOVA and the Tukey–Kramer post hoc test. In all panels, the asterisks indicate significant values as follows: *P < 0.05; **P < 0.01; and ***P < 0.001. For data, see Supplementary Table 1.

Because the *Tbh*^*nM18*^ mutation did not delete the translational start site of *Tbh*^*RA*^ and *Tbh*^*RB*^ and we detected *Tbh* transcripts in *Tbh*^*nM18*^ mutants, we decided to generate a new mutant with a missing translation start site by FLP/FRT recombination-based mutagenesis^18^. Transheterozygous flies carrying the FRT site containing transposons *XP*^*d01344*^ upstream and *XP*^*d10000*^ downstream of the second exon of the *Tbh* gene were used as starter lines (Fig. 2A). Activation of FLP recombinase in these flies resulted in the removal of the genomic sequence between the two transposons and fragments of the transposable elements. The newly generated *Tbh*^*Del3*^ allele contained a 9.2 kb deletion, including the first and second exons of *Tbh*, as confirmed by PCR analysis (Fig. 2A). The deletion affected *Tbh*^*RA*^ to *Tbh*^*RC*^ but not *Tbh*^*RD*^.

Next, we used qRT‒PCR to analyze whether the new deletion changed the *Tbh* transcript level in the head of the flies. (Fig. 2B). We used the parental lines for comparison. In the *Tbh*^*Del3*^ mutants, the level of the *Tbh*^5-^^6^ transcript fragment was reduced by approximately 83%, and that in the insertion line *XP*^*d10000*^ was substantially reduced by 68%. In contrast, in the *XP*^*d01344*^ insertion line, the amount of the *Tbh*^5-^^6^ transcript fragment significantly increased by approximately 62%. Because the *Tbh* transcript levels were altered in the *XP*^*d01344*^ and *XP*^*d10000*^ lines, the lines were considered *Tbh* mutants.

*Tbh*^*nM18*^ mutants develop reduced ethanol tolerance^10^. To determine whether the *Tbh*^*Del3*^ mutants also had reduced ethanol tolerance, we analyzed the ethanol sensitivity and tolerance of the *Tbh*^*Del3*^ mutants and the parental insertion lines (Fig. 2C). To measure ethanol sensitivity and tolerance, the flies were inserted into the “inebriometer”, a device for measuring the effect of ethanol on postural control in a population of flies^19^. After a first exposure and a second exposure four h later, flies typically show an increased resistance to the effect of ethanol on postural control. This relative increase is defined as ethanol tolerance^10^. To quantify this behavior, the average time a population spent in the column is determined as the *mean elution time* (MET). The ethanol sensitivity of *Tbh*^*Del3*^ and *Tbh*^*01344*^ flies did not differ from that of the controls, but the resistance of the *Tbh*^*d10000*^ flies was significantly increased approximately 11% (Fig. 2C). The control flies developed an ethanol tolerance of approximately 25%, similar to that of *Tbh*^*d01344*^ flies. In contrast, the tolerance of the *Tbh*^*Del3*^ mutants significantly decreased by approximately 64%, and that of the *Tbh*^*d10000*^ mutants significantly decreased by approximately 55% (Fig. 2C). *Tbh*^*Del3*^ and *Tbh*^*d10000*^ mutants exhibited a reduced tolerance phenotype similar to that of the *Tbh*^*nM18*^ mutants and failed to complement the female sterility of the *Tbh*^*nM18*^ mutants.

*Tbh*^*nM18*^ mutants are more resistant to ethanol when exposed to 30 min of heat shock at 37 °C four h before ethanol exposure, similar to control flies^14^. To determine whether the new *Tbh*^*Del3*^ mutant also develops heat-induced resistance to ethanol, we analyzed the ethanol-induced behavior of *Tbh*^*Del3*^, *Tbh*^*nM18*^ mutants and control flies after heat shock (Fig. 2D). In *Tbh*^*nM18*^ mutants, prior heat exposure resulted in an approximately 22% increased resistance to ethanol compared with the ethanol-induced behavior of mutant flies not exposed to heat. This increase in ethanol resistance was comparable to that of control flies. In contrast, the resistance of the *Tbh*^*Del3*^ mutant flies to ethanol significantly decreased by approximately 35% after heat shock (Fig. 2D). In conclusion, the *Tbh*^*10000*^ mutant was more resistant to ethanol, developed tolerance comparable to that of the control, and exhibited increased *Tbh* transcript levels. The new *Tbh*^*Del3*^ mutants, like the *Tbh*^*nM18*^ mutants, exhibited reduced ethanol tolerance and *Tbh* transcript levels but differed in their resistance to ethanol after heat shock.

### *Tbh* mutants respond to external stimuli and changes in their internal condition with increased locomotion

**Fig. 3:**
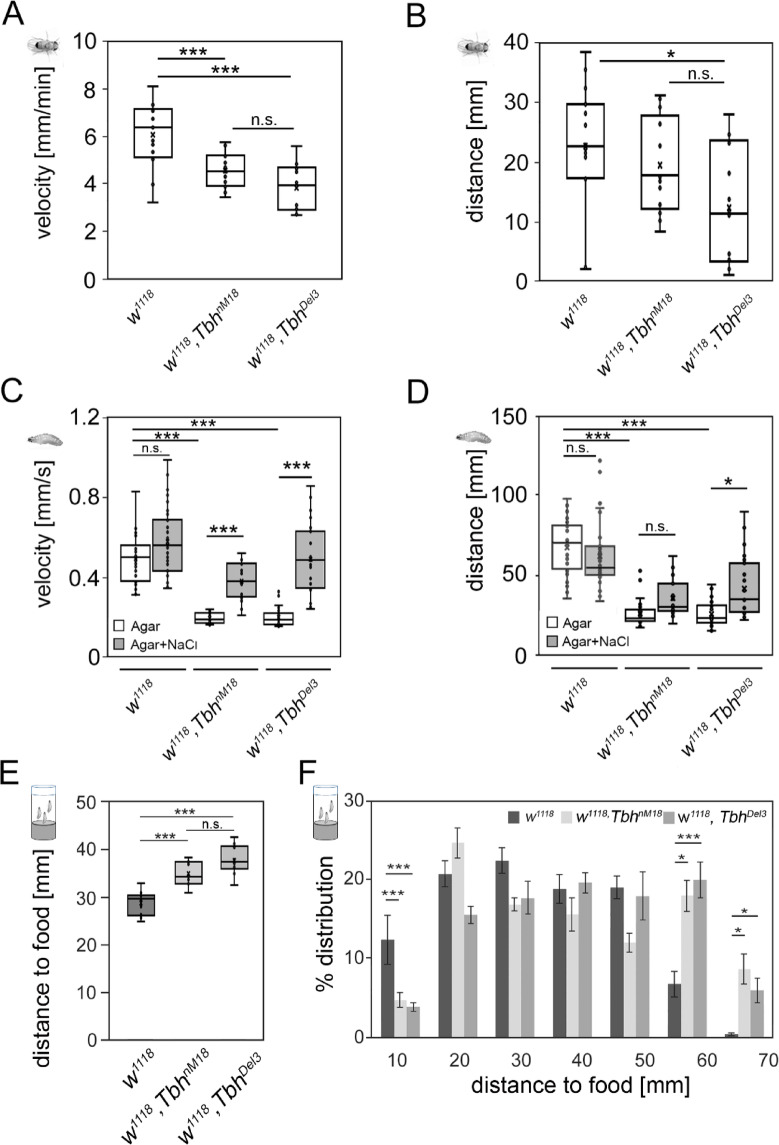
Reduced movement of *Tbh* mutants is improved by external stimulation and internal motivation. The speed (**A**) and distance traveled (**B**) of individual male flies were analyzed in a glass arena. Compared with the controls, the *Tbh*^nM18^ and* Tbh*^Del3^ mutants walked significantly slower (ANOVA; F (2,43) = 16,98; *P* = 0.0000037), and the *Tbh*^Del3^ mutants walked less quickly (ANOVA (F (2,43) = 4,85; *P* = 0.013) (N = 11–13 flies). (**C**) The *Tbh* mutant larvae moved significantly slower but faster when they were exposed to salt. (**D**) Similarly, the Tbh mutant larvae traveled a significantly shorter distance, and the distance traveled by the *Tbh*^Del3^ mutants significantly increased when they were exposed to 2 M NaCl salt (N = 11–13 larvae per genotype). (**E**) Compared with the control larvae, the 3rd-instar *Tbh* mutant larvae moved significantly farther from the food to pupate (ANOVA (F(2,26) = 29,4662; *P* = 0.0000002073). (**F**) Analysis of the distribution pattern of the distances traveled collected in 10 mm bins revealed that the *Tbh*^nM18^ and *TbhDel3* larvae moved significantly farther from the food to pupate (N = 9–10 vials with at least 50 pupae per vial). Normal distributed data were compared by ANOVA with a Tukey–Kramer post hoc test. The asterisks indicate significance as follows: *P < 0.05 and ***P < 0.001 for (**A**) and (**B**). For nonnormally distributed data in (**C**) and (**D**), the Kruskal‒Wallis test followed by a Bonferroni correction (α = 0.003333) was used to determine differences. *P < 0.0033; and **P < 0.00066 and ***P < 0.00033 for (**C**) and (**D**). For data, see Supplementary Table 1.

*Tbh*^*nM18*^ and *Tbh*^*Del3*^ mutants exhibited normal ethanol sensitivity, which indicates that they had the motor skills to perform the task required in a behavioral assay. However, they failed to adjust their behavioral output after a second ethanol exposure. To further investigate motor performance, we analyzed how well adult and larval *Tbh*^*nM18*^ and *Tbh*^*Del3*^ mutants move (Fig. 3). We filmed the flies for one min and analyzed their walking trajectories (Fig. 3A and B). *Tbh*^*nM18*^ flies moved significantly slower than control flies did by approximately 19%, and *Tbh*^*Del3*^ flies moved slower than control flies by approximately 28% (Fig. 3A). Additionally, the distance traveled by *Tbh*^*nM18*^ flies was similar to that traveled by control flies, whereas the distance traveled by *Tbh*^*Del3*^ flies was approximately 35% shorter (Fig. 3B).

To examine the movement of the larvae, control and *Tbh* mutant larvae were placed on agar plates, and their movements were monitored for two min. Consistent with previous results, *Tbh*^*nM18*^ mutant larvae moved at significantly reduced speeds and crawled shorter distances^20,21^ (Fig. 3C and D). *Tbh*^*Del3*^ mutants behaved similarly (Fig. 3C and D).

The locomotion and speed of adult *Tbh*^nM18^ mutants increased when they were exposed to ethanol, suggesting that the environmental stimulus and/or the motivation to move were not strong enough to elicit the same locomotor pattern in the absence of ethanol^5^. To encourage the movement of *Tbh*^*nM18*^ mutant larvae, we exposed them to an aversive stimulus by adding 2 M NaCl to the agar plate (Fig. 3C and D). The control larvae did not significantly increase their movement speed or distance traveled. *Tbh*^*nM18*^ mutants increased their speed twofold, and *Tbh*^*Del3*^ mutants moved 2.5 times faster and crawled 68% farther.

To analyze whether intrinsic motivation influences movement patterns, we measured the distance that 3rd -instar larvae crawl out of food to pupate (Fig. 3E and F). *Tbh*^*nM18*^ and*Tbh*^*Del3*^ mutant larvae crawled 22–31% farther out of food to pupate than control larvae did (Fig. 3E). Analysis of the distribution pattern of the distance traveled revealed that the *Tbh*^*nM18*^ and *Tbh*^*Del3*^ larvae traveled significantly longer distances from food to pupate (Fig. 3F). Therefore, 3rd -instar larvae of the *Tbh* mutant larvae had the ability to cover longer distances. Taken together, the observations that *Tbh* mutants developed normal ethanol sensitivity and walked as quickly and at least as long as controls when exposed to salt suggest that *Tbh* mutants did not have defects in their locomotor ability. Rather, they exhibited deficiencies in translating a previous experience into a new behavior and had defects in adapting their behavior in response to changes in the external and internal environment.

### Tbh is required in adult flies for ethanol tolerance

**Fig. 4:**
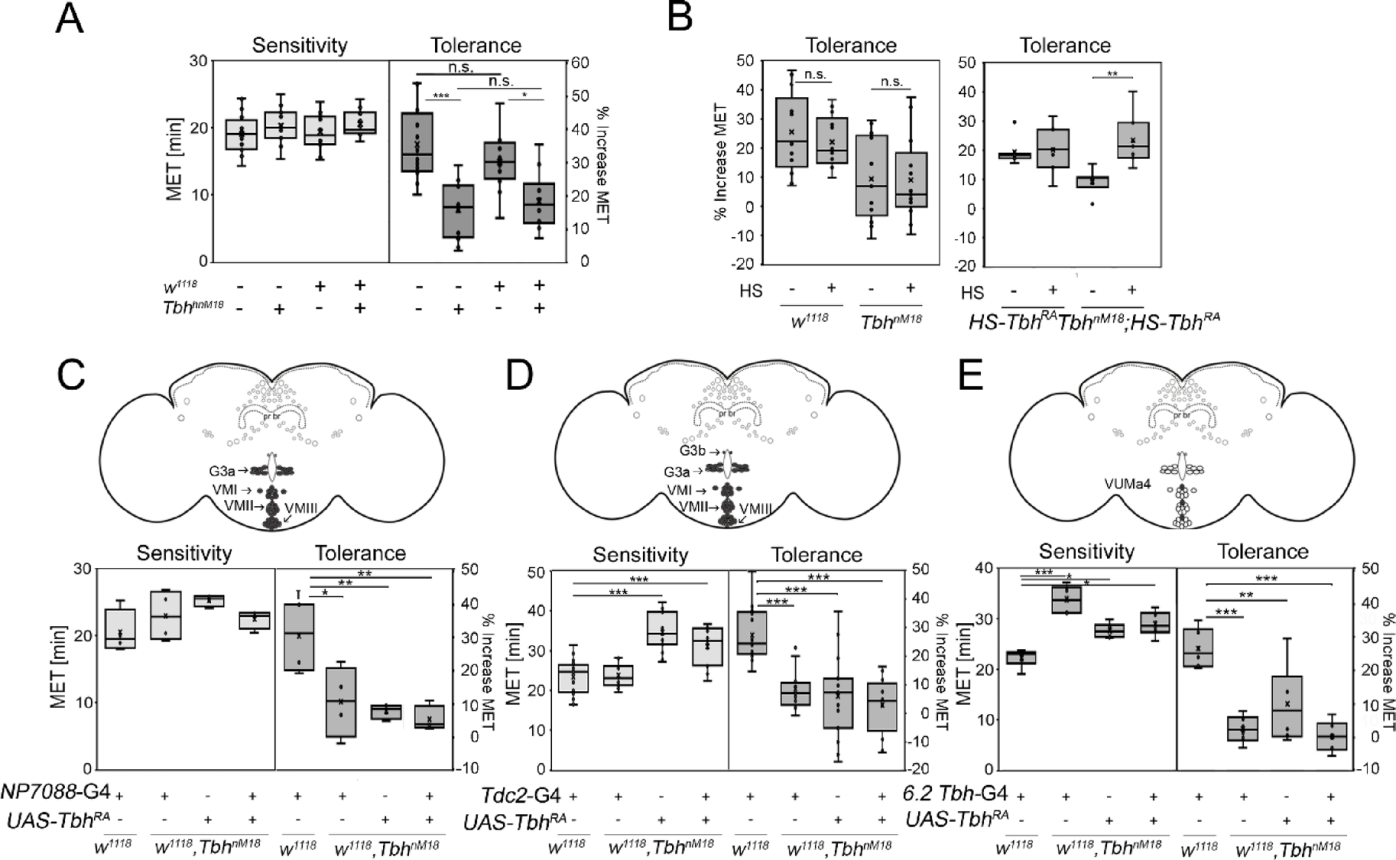
Tbh is required for ethanol tolerance in adult flies. (**A**) The *w*^1118^ mutation did not influence ethanol sensitivity or reduce the ethanol tolerance of *Tbh*^nM18^ mutants (N = 10–16) (ANOVA sensitivity F (3,46) = 0,8113, *P* = 0.4942; ANOVA tolerance F (3,46) = 13.5581, *P* = 0.000002, with *P* = 0.4256 for w1118 versus w+ and *P* = 0.9247 for *w*^1118^ Tbh^nM18^ versus *w*^+^Tbh^nM18^). (**B**) Controls and *Tbh*^nM18^ mutants received a 30 min heat shock of 37° 16 h before the first ethanol exposure. Heat shock had no effect on tolerance in the controls or on the reduced tolerance of *Tbh*^nM18^ mutants (N = 13) (ANOVA tolerance F (3,49) = 4.7866, *P* = 0.005287, with *P* = 0.9998 for *w*^1118^ Tbh^nM18^ versus *w*^1118^ Tbh^nM18^ +HS). The reduced tolerance of the *Tbh*^nM18^ mutants was restored to normal levels by induction of the hs-*Tbh*-*RA* transgene (N = 5–7) (ANOVA tolerance F (3,24) = 5.8236, *P* = 0.003878, with *P* = 0.003111 for* w*^1118^ Tbh^nM18^, HS-*Tbh*-*RA* versus *w*^1118^ Tbh^nM18^, HS-*Tbh-RA* +HS). The expression of *Tbh-RA* under the control of (**C**) *NP7088*-Gal4 (N = 4) (ANOVA F (3,12) = 8.0784, *P* = 0.003273, with *P* = 0.004278 for the control with normal tolerance group versus the experimental group), (**D**) *dTdc2*-Gal4 (N = 12–17) (ANOVA F (3,52) = 16.4332, *P* = 1.225e-7, with *P* = 9.462e-7 for the control with normal tolerance group versus the experimental group) or (**E**) the 6.2-*Tbh*-Gal4 driver (N = 6) (ANOVA F (3,20) = 16.4879, P = 0.00001244, with *P* = 0.00001976 for the control with normal tolerance group versus the experimental group) did not restore the reduced ethanol tolerance of the *Tbh*^nM18^ mutants. In the schemes, Tbh-positive neurons that are addressed by the respective Gal driver are shown in gray filled circles. ANOVA with the Tukey–Kramer post hoc test was used to determine differences between groups. Nonsignificant differences are indicated as n.s., and the asterisks indicate *P < 0.05 and **P < 0.01. For data, see Supplementary Table 1.

To determine at which developmental stage Tbh is required for ethanol tolerance, we wanted to restore Tbh expression in the *Tbh*^*nM18*^ mutants at the adult stage with a heat-inducible *Tbh* transgene. First, we analyzed whether the *white* gene, which is used as a marker for P-element insertion of the transgenes, influences ethanol sensitivity and the development of ethanol tolerance. Therefore, we tested the ethanol sensitivity and tolerance of *w*^+^, *Tbh*^*nM18*^ and *w*^−^, *Tbh*^*nM18*^ and their respective controls (Fig. 4A). Both *w*^*+*^, *Tbh* ^*nM18*^ and *w*^*1118*^, *Tbh*^*nM18*^ mutants developed normal ethanol sensitivity and a similar significantly reduced tolerance by approximately 40 to 55%. Thus, the *white* mutation had no effect on the sensitivity or reduced tolerance of the *Tbh* mutants.

We previously showed that heat shock at 37 °C for four h before ethanol exposure increased the resistance to ethanol^14^. To avoid the possible influence of 37 °C heat shock on ethanol tolerance, heat shock was applied 16 h before the first ethanol exposure. As an additional control, we analyzed the effect of heat shock treatment on the development of ethanol tolerance in controls and *Tbh*^*nM18*^ mutants (Fig. 4B, left). Heat treatment 16 h before ethanol exposure had no effect on the tolerance of the controls or the reduced tolerance of the *Tbh*^*nM18*^mutants. Next, we used the same procedure to induce the *hs*-*Tbh*^*RA*^ transgene in the *Tbh*^*nM18*^ mutants (Fig. 4B, right). In the controls, heat shock had no effect on tolerance, but the reduced tolerance phenotype of the *Tbh*^*nM18*^ mutants significantly improved to control levels (Fig. 4B, right). These findings led us to conclude that Tbh is required for ethanol tolerance in the adult stage.

To identify in which neurons *Tbh* is required for tolerance, we restored Tbh expression in the *Tbh*^*nM18*^ mutants with different Gal4 drivers. The Gal4 lines drove transgene expression in subsets of Tbh-positive neurons (Fig. 4C and E;^12^). Tbh expression under the control of the tyraminergic/octopaminergic *dTdc2*-Gal4 and *NP7088*-Gal4 driver lines^22^ and the *6.2-Tbh*-Gal4 driver^12^ did not significantly improve the reduced ethanol tolerance of *Tbh*^*nM18*^ mutants (Fig. 4C and E). Thus, none of the Gal4-targeted Tbh-positive neurons, including ventral unpaired median neurons (VUMs) and G3a or G3b neurons, appeared to regulate Tbh-dependent ethanol tolerance. These findings distinguish the neurons mediating Tbh-dependent ethanol preference^12^ from those responsible for experience-dependent ethanol tolerance.

### A subset of neurons mediates Tbh-dependent ethanol tolerance

**Fig. 5:**
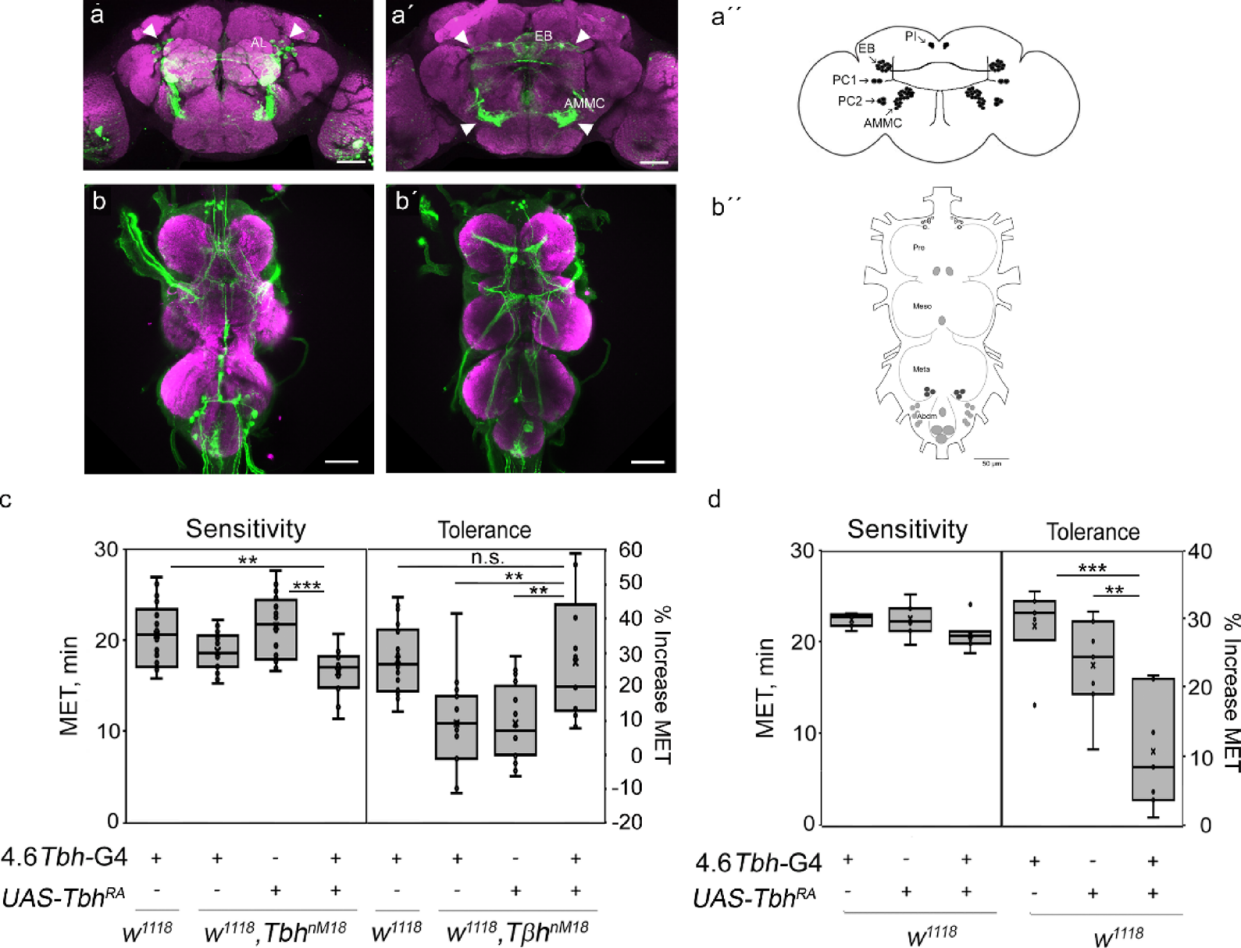
Tbh expression under the control of *4.6*-*Tbh*-Gal4 restores the reduced tolerance to control levels. The Gal4 expression pattern of the *4.6-Tbh*-Gal4 driver was visualized with a *UAS-mCD8*:GFP transgene (in green), and the neurons were visualized with the marker nC82 (in magenta) in the brain (**a**) and the ventral nerve cord (**b**). The anterior (**a**) and posterior (**a´**) parts of the brain are shown. The antennal lobe (AL), ellipsoid body (EB), and antennal-mechanosensory and motor center (AMMC) are labeled, and the white arrowheads indicate the soma or projections. In (**b**), the ventral and (**b´**) dorsal parts of the VNC are shown. The cartoons in (**a´´**) and (**b´´**) summarize the GFP-positive neurons as dots. (**c**) The expression of *Tbh* in a *4.6-Tbh*-Gal4-dependent manner restored the reduced tolerance of *Tbh*^*nM18*^ mutants to control levels (*N* = 14–18) (ANOVA sensitivity F(3,62) = 7.9216, *P* = 0.0001593, with *P* = 0.002496 for the Gal4 driver group compared to the experimental group and *P* = 0.00017 for the *UAS*-*Tbh*^*RA*^ transgene in the mutant background group compared to the experimental group; ANOVA tolerance F(3,62) = 9.4166, *P* = 0.000036, with *p* = 0.004 for the Gal4 driver in the mutant background group compared to the experimental group and *P* = 0.003953 for the UAS transgene in the mutant background group compared to the experimental group). (**d**) Overexpression of *Tbh* with the *4.6-Tbh*-Gal4 driver significantly reduced ethanol tolerance (*N* = 7) (ANOVA sensitivity F (2,20) = 27.523, *P* = 0.09059; ANOVA tolerance F (2,20), *P* = 0.000415, with *P* = 0.00035 for the Gal4 driver group compared to the experimental group and *P* = 0.009562 for the UAS transgene group compared to the experimental group). The asterisks represent **P < 0.01 and ***P < 0.001; for data, see Supplementary Table 1.

Because the *dTdc2*-Gal4 and 6.2-*Tbh*-Gal4 drivers do not target the entire set of Tbh-positive neurons^12^, we generated a new *Tbh-*promoter-Gal4 driver. For this purpose, we used a 4.6 kb fragment spanning the genomic region − 877 to + 3718 of the *Tbh* gene as a promoter fragment (indicated in Fig. 1A) and characterized the expression pattern of the *4.6-Tbh*-Gal4 driver in the brains of adult flies with the *UAS-mcD8::GFP* transgene (Fig. 5A). GFP expression was detected in approximately 61–72 cells (Fig. 5A and A´´). In each hemisphere, the following clusters were identified: a cluster of 11 to 13 cells innervating the ellipsoid body (EB), a cluster of 16 to 18 cells innervating the antennal lobes (AL), and one cell localized in the pars intercerebralis (PI) projecting toward the esophagus. In addition, there are two clusters in the posterior part of the brain: one cluster with two cells (PC1) and a second cluster with two to three cells (PC2). Strong Johnston’s Organ expression was also found projecting into the antennal–mechanosensory and motor center (AMMC) (Fig. 5A´). In the ventral nerve cord, GFP-expressing neurons were found in the pro-, meso- and metathorax and in the abdominal segments (Fig. 5B and B´´). None of the targeted 4.6 Tbh-Gal4 neurons showed detectable octopamine levels (Supplementary Fig. 1). This appears at the first glance puzzling. However, octopamine has a very high turn-over rate^23^. In addition, octopamine is difficult to reliably visualize. For example, immunoreactivity is largely restricted to the soma and often undetectable unless animals are cooled prior to tissue preparation (own observation; see also^22,24^). Importantly, these technical limitations do not affect our conclusions, as the functional role of Tbh in these neurons can be assessed independently of detectable octopamine levels. The expression of *UAS-Tbh*^*RA*^ under the control of the *4.6-Tbh-*Gal4 driver restored the reduced ethanol tolerance of *Tbh*^*nM18*^ to control levels (Fig. 5C). To determine whether the dose of the Tbh enzyme is important for normal ethanol tolerance development, we overexpressed Tbh in control flies with the *4.6-Tbh*-Gal4 driver (Fig. 5D). The increased levels of *Tbh* significantly reduced ethanol tolerance, suggesting that the Tbh level needs to be tightly regulated for tolerance to develop.

## Discussion

The *Tbh* gene encodes at least four transcripts and three different isoforms whose sizes differ. Two of the identified transcripts differ in their 5′-UTRs and have the same coding sequences, suggesting that their translation is differentially regulated. At the protein level, the different isoforms differ in the number of six putative protein kinase C phosphorylation sites. For example, the isoform Tbh^PC^ contains six putative protein kinase C phosphorylation sites, whereas Tbh^PD^ has only two sites. These differences may lead to changes in Tbh enzyme expression and activity. Changes in Tbh activity have been observed during development and upon exposure to stress. In the moth *Manduca sexta*, the activity of Tbh increases during adult development^25^, and in the cockroach *Periplaneta americana*, the activity of Tbh increases shortly after mechanical stress stimulation^16^. The newly generated *Tbh*^*Del3*^ mutants exhibited similar locomotor and ethanol tolerance defects as the *Tbh*^*nM18*^ mutants did. The reduced locomotion of *Tbh* mutants was due to a lack of adaptation in motor performance rather than defects in behavioral execution, since providing a strong environmental stimulus could increase locomotion. Similarly, *t*hey developed reduced tolerance after previous ethanol exposure but eventually reached the same level after multiple ethanol doses or with chronic ethanol administration^10,15^. The development of tolerance is an adaptation to previous experience. The observation that the behavioral defects in *Tbh* mutants could be modulated by external stimuli or changes in internal conditions suggests that the octopaminergic system affects the behavioral outcome^13^.

The inability to properly regulate the development of ethanol tolerance was traced to neurons targeted by the *4.6*-Gal4 driver. Neurons that normally regulate Tbh-dependent innate ethanol preference—including VUM and G3a or G3b neurons—can be excluded from the control of experience-dependent ethanol tolerance, as the *dTdc2*-Gal4 driver failed to restore the reduced ethanol tolerance observed in the *Tbh* mutant.Our findings reveal a functional dissociation between the neuronal circuits regulating innate ethanol preference and those mediating ethanol tolerance. While Tbh in *dTdc2*-GAL4 neurons is critical for ethanol preference, tolerance relies on separate pathways, highlighting that molecular and circuit-level mechanisms underlying ethanol-related behaviors are behavior-specific. One brain region involved in the regulation of ethanol tolerance is the central complex^10,26^. A pair of F1 neurons within the central complex mediate Dunce-dependent ethanol tolerance and experience-dependent changes in locomotion^27,28^. Whether and how Tbh-positive neurons located in the posterior part of the brain are connected to the central complex remain to be determined. In addition, they might be other candidate neurons in the VNC that may regulate ethanol tolerance.

The *Tbh*^*Del3*^ mutants differed from *Tbh*^*nM18*^ in their response to heat and ethanol. *Tbh*^*Del3*^ mutants responded less strongly to heat-induced ethanol resistance, whereas *Tbh*^*nM18*^ mutants responded normally. The stress dependence of Tbh function was also indirectly demonstrated by changes in octopamine levels. In *Drosophila virilis*, a close relative of *D. melanogaster*, heat exposure increases octopamine levels^29^. In the locust *Schistocerca gregaria*, mechanical stress leads to the conversion of tyramine to octopamine, resulting in an increase in octopamine levels^30^. These results are consistent with the assumption that stress-inducible and nonstress-inducible octopamine synthesis occur. These differences could be regulated by different *Tbh* transcripts.

In summary, Tbh function can be controlled at the transcriptional and protein levels. These mechanisms allow for the temporary control of Tbh functions in response to changes induced by, for example, environmental stressors such as heat. The presence of different Tbh isoforms and the structural similarities between Dbh and Tbh suggest that it is also possible that Tbh could synthesize other neurotransmitters.

## Methods

### Fly stocks

The following lines were used: *Tbh*^*nM18*1^, *dTdc2*-Gal4^31^, NP7088-Gal4^22^, *6.2*-*Tbh*-Gal4^12^, *hs-Tbh*^4^, *UAS*-*Tbh*^*RA*12^, and *UAS*-mCD8::GFP^32^. The lines *XP*^*d10000*^ and *XP*^*d01344*^ were obtained from Exelixis at Harvard Medical School. Flies carrying transposable elements were backcrossed to the *w*^*1118*^ background in the laboratory for at least five generations. Flies were reared on standard ethanol-free media with a 12–12 h light‒dark cycle at 25 °C and 60% humidity under density-controlled conditions. For the behavioral experiments, three- to five-day-old male flies that had recovered from CO_2_ sedation for 36 h were used.

### Ethics declarations

The animals used for the experiments were *D. melanogaster*. The experiments were conducted under S1 safety measures and in accordance with the Animal Welfare Act of the State of North Rhine-Westphalia.

### Generation of theTbh^*Del3*^ allele

FLP/FRT recombination was performed as previously described^18^. The *XP*^*d10000*^ and *XP*^*d01344*^ lines were used as parental FRT sites carrying P-element insertion lines (obtained from the Exelixis Collection at Harvard Medical School). In transheterozygous flies carrying both XP lines, recombination was induced with a heat shock-inducible flipase, resulting in a deletion of the sequence between the two FRT sites of the transposons. To generate a deletion, the following crosses were established. Specifically, 35 homozygous *XP*^*d01344*^ virgin females were mated with 15 male flies carrying the *hs*-FLP transgene on the third chromosome (*w*^*1118*^; MKRS, P{ry[+ t7.2]=hsFLP}86E/TM6B, Tb^1^ - BDSC #279). The crosses were set up 30 times. Subsequently, 45 crosses with 15 males of the F1 generation were mated with 35 *XP*^*d10000*^*/FM7* virgin females. After three days, the parental flies were removed, and the larvae were exposed to heat shock in a 37 °C water bath for one h on four consecutive days. In the next generation, 35 virgin females of the F2 generation were mated to 15 males carrying the first chromosomal balancer, Binsinscy (BDSC #7759). In the F3 generation, 350 individual male flies were mated to three to five *Tbh*^*nM18*^/FM7 virgin females to establish a stock and to select for female sterility. Next, males of the newly generated stocks, which were also female sterile, were screened for genomic deletion by PCR analysis.

First, the 5′ and 3′ flanking sequences associated with XP insertion lines were confirmed. To map the *XP*^*d01344*^ insertion, the primers 5′–TGGCACACACTTACGGGTTA-3′ and 5′-GGGAAACGCCTGGTATCTTT-3′ were used, and for the *XP*^*d01000*^ insertion, the primers 5′-GTGCAAAGTGCTCACGCTTA-3′ and 5′–GGGAAACGCCTGGTATCTTT-3′ were used. The deletion contains only fragments of the *XP*^*d01344*^ insertion. The following primers were used to map the truncated *XP*^*d10000*^ P-element insertion: 5′-TTAGCTGCACATCGTCGAAC3′ and 5′-AGCCGGATGACATTATCTGC-3′. The deletion was confirmed with the following primers: 5′-ATTCCGCTGCAGCTGAGCAG-3′ and 5′-GGACTGACACTCACGGAGACA-3′. Among the approximately 40 female sterile lines, 5 lines carried the deletion. Before their use, the newly generated *Tbh*^*Del3*^ mutant and the parental XP-element insertion lines were backcrossed with *w*^*1118*^ for five generations to isogenize the background.

### Identification of *Tbh* transcripts

To analyze *Tbh* transcripts, we isolated total RNA from three- to five-day-old adult *w*^*1118*^ fly heads with TRIzol™ reagent (Invitrogen). cDNA was generated with oligo-dT primers and SuperScript™ II reverse transcriptase. To identify additional sequences at the 5′ end, we analyzed the sequences of the *Tbh* EST clones. The cDNA clone EY198604.1 contained an additional sequence at the 5′ end of the primer. The sequence was confirmed using the 5′ prime end-specific 5′-ACGCGCTTTCCACTTGTTCG-3′ primer. To identify additional transcripts, we used primers that recognize sequences in the 5′ prime region combined with primers that recognize sequences in the 3′ prime region to amplify splice variants (the 5′ end primer 5′-ACGCGCTTTCCACTTGTTCG-3′ combined with the 3′ end primer 5′-GCTTTCGCTTGGTTTTTGTT-3′). Exon 3 was identified by analyzing putative open reading frames in intron three and designing primers against the sequence. To validate the new exon 3, polyA-selected RNA was isolated from the total RNA of 1000 heads of one- to two-day-old flies with the MicroPoly(A)Purist™ Kit (Ambion). After cDNA synthesis with oligo-dT primers, nested PCR was performed with LongAmp^®^ *Taq* DNA Polymerase. For the first synthesis, the following primers were used: 5′-CGAGTGCGATGCATCAAGTG-3′ and 5′-CCATTCGATGCTCCGGTAAT-3′. For the second synthesis, the following primers were used: 5′-CCCAAAAGGGTCGTTCTGTC-3′ and 5′-CAGTTTGGTGGCCGGATAG-3′. An isolated fragment of 1.5 kb was subcloned and sequenced.

### Quantitative real-time PCR

Total RNA was isolated from fly heads by acid guanidinium thiocyanate–phenol–chloroform extraction with TRIzol^®^ reagent (Invitrogen). For cDNA synthesis, we used oligo-dT primers and SuperScript™ II reverse transcriptase. Control primers were selected with NormFinder software^33^. The level of the *Tbh* transcripts was normalized to the transcription of the reference gene *RpLP0* (5′-CAGCGTGGAAGGCTCAGTA-3′ and 5′-CAGGCTGGTACGGATGTTCT-3′). Melt curve analysis was used to test for off-target amplification, and gel electrophoresis was performed to analyze the PCR products and confirm their size. The linear dynamic range of the qRT‒PCR was determined by serial dilution of the cDNA and analysis of the threshold cycle values in relation to the dilution factor. The ΔΔCt method was used to determine relative transcript levels (^34^). Experiments were carried out with the iCycler iQ5 Multicolour Real-Time PCR Detection System and the accompanying iQ5 Optical System Software from Bio-Rad. The data are shown as fold changes in *Tbh* transcription relative to the reference primers. The primers used to detect the *Tbh*^5-^^6^ region were 5′-ATCCGTACGTTCGACTGGAG-3′ and 5′-TCGACATCTTGATGCGAAAG-3′. The primers used to detect the induction of *hsp70* transcription were 5′-GGCATATCTGGGCGAGAGCATC-3′ and 5′-CTTGAACTCGTCCGCCAGATGAG, and those for *Rap2l* were 5′-ACTTCCGTGCATTACGTGCG-3′ and 5′-CCGACCCGAGCACAACAACT-3.

### Generation of the 4.6-Tbh-Gal4 driver

The 4.6 kb promoter fragment of the *Tbh* gene, ranging from − 877 to + 3718, was amplified with the primers 5′-TGGCACACACTTACGGGTTA-3′ and 5′-GGTGGTGATGGAGTCGCC-3′. The fragment was subsequently cloned and inserted into the p221-4 GAL4 vector via the pCRII vector (Life Technologies) with BamHI and NotI restriction sites. The construct was injected into *w*^*1118*^ embryos to generate transgenic lines. We used random insertions to generate lines with different Gal4 expression patterns and different chromosomal insertions. Insertion #6.5 on the second chromosome was chosen for the experiments.

### Behavioral assays

Sensitivity and tolerance to ethanol were tested as previously described^19,10^. Briefly, 120 flies were introduced into a column filled with ethanol vapor. The average time a population spent in the column, defined as the *mean elution time* (MET), was determined. MET1 reflects the initial sensitivity to the effect of ethanol on postural control, whereas MET2 refers to the second exposure to ethanol^19,10^. After the first exposure, the flies were collected and reinserted into the columns after a recovery period of four h. The tolerance was calculated as follows: (MET2–MET1)/MET1*100. To determine the effect of heat shock on ethanol-induced behavior, 100 flies in an empty vial were incubated in a water bath at 37 °C for 30 min, followed by a 3.5 h recovery at 25 °C. The flies were then exposed to ethanol in the inebriometer (MET + hs). Tolerance was calculated in comparison to that of non-heat-treated flies (MET-hs).

For the behavioral analysis, the larval density was controlled by crossing 35 virgin females with 17 males. The flies were allowed to lay eggs for 24 h at 25 °C and 65% humidity. Wandering 3rd -instar larvae of both sexes were used for the experiments. *Tbh* mutant larvae were collected under fluorescent light to exclude larvae carrying the FM7-GFP balancer. The experiments were repeated with different groups of larvae at least three times on three different days.

Frustrated total internal reflection-based imaging methods (FIM; ^35^) were used to analyze behavior. Up to five larvae were placed on an FIM table covered with 0.8% agarose gel with or without 2 M NaCl. Behavior was recorded for 2 min. The trajectory of a single larva was analyzed with FIM Track software^35^.

To analyze the trajectory of a single adult fly, a 3- to 5 days old male was placed in a 3.6 cm diameter glass Petri dish. The dish was illuminated from below with a cold light source and enclosed in a dark box. The fly was allowed to rest for 5 min in a Petri dish to acclimate to the environment. Its movement was subsequently recorded for 1 min at 25 frames per s. The video was analyzed with the TrackMate plugin of ImageJ.

To measure the distance traveled by flies from food to pupation, 12 *w*^*1118*^ virgin females were crossed with 4 *w*^*1118*^ males, and 48 heterozygous *Tbh* mutants with balancer FM7-GFP females were crossed with 16 hemizygous *Tbh* males. After two days of egg laying, the adult flies were removed from the vial. Eight days later, the distance between the food surface and the center of each pupa was measured. Pupae harboring the FM7-GFP balancer were selected under green fluorescent light and excluded from analysis. The data show the mean and distribution of food-to-pupae distances per vial.

To measure food intake, a group of 10 adult male flies, aged 2–3 days, were collected and transferred to standard food for 24 h at 25 °C. Prior to being transferred into vials containing 5% sucrose in 1% agarose and 0.5% food dye, the flies were starved for 18 h at 25 °C in a vial with humidified filter paper but no food. Food intake was assessed by scoring abdomen coloration as previously described^36^. To minimize the potential effects of circadian rhythms on feeding behavior, the flies were starved from 4 PM to 10 AM the following day.

### Immunohistochemical staining

The brains of three- to eight-day-old male flies were used for immunohistochemical staining. The dissection, fixation and staining of the nervous system were performed as previously described^12^. The following antibodies were used: mouse anti-nc82 (1:20, DSHB), rabbit anti-GFP (1:1000, Invitrogen), Alexa Fluor 488-conjugated anti-nc82 goat anti-rabbit (1:1000, Invitrogen), and Cy3-conjugated goat anti-rabbit IgG (Jackson ImmunoResearch, 1:200). Optical sections of one µm were generated with a Zeiss LSM 510 laser scanning microscope. The data analysis of the confocal stacks was performed with Fiji (ImageJ).

The strains and plasmids are available upon request. The authors certify that all the data necessary to confirm the article’s conclusions are included in the article, figures and supporting information.

### Statistical analysis

The Shapiro–Wilk test (significance level *P* < 0.05) followed by a QQ-Plot chart analysis was used to analyze whether the data were normally distributed. For normally distributed data, significance was determined by one-way ANOVA combined with the Tukey–Kramer *post hoc* HSD test. For the qPCR analysis, a two-sample T test (Welch) was used. For nonparametric data, the Mann‒Whitney U test for differences between two groups and the Kruskal‒Wallis test with post hoc Dunn analysis and Bonferroni correction for differences among more than two groups were used. Statistical data analysis was performed with statskingdom (https://www.statskingdom.com). The errors are indicated as the standard error of the mean (s.e.m.).

## Supplementary Information

Below is the link to the electronic supplementary material.

Supplementary Material 1

Supplementary Material 2

## Data Availability

Data from the behavioral experiments are included in Supplementary Table 1. The sequences of Tbh transcripts and Tbh proteins identified in the current study are available in Genbank Accession numbers as follows: SUB16040049 Dmel_CG1543_headcDNA_Tbh-RA PZ137124,SUB16040049 Dmel_CG1543_headcDNA_Tbh-RB PZ137125,SUB16040049 Dmel_CG1543_headcDNA_Tbh-RC PZ137126, andSUB16040049 Dmel_CG1543_headcDNA_Tbh-RD PZ137127.SUB16040049 Dmel\_CG1543\_headcDNA\_Tbh-RB\t PZ137125,SUB16040049 Dmel\_CG1543\_headcDNA\_Tbh-RC PZ137126, andSUB16040049 Dmel\_CG1543\_headcDNA\_Tbh-RD\t PZ137127.
