# Supplementary material for: The Drosophila tyramine beta-hydroxylase gene is required for ethanol tolerance
Source: Sci Rep. 2026 Apr 11;16:12180. doi: 10.1038/s41598-026-45082-3 (PMC13076892; doi:10.1038/s41598-026-45082-3)
Supplement: Supplementary file 2 — Supplementary Material 2 [file 41598_2026_45082_MOESM2_ESM.pdf]

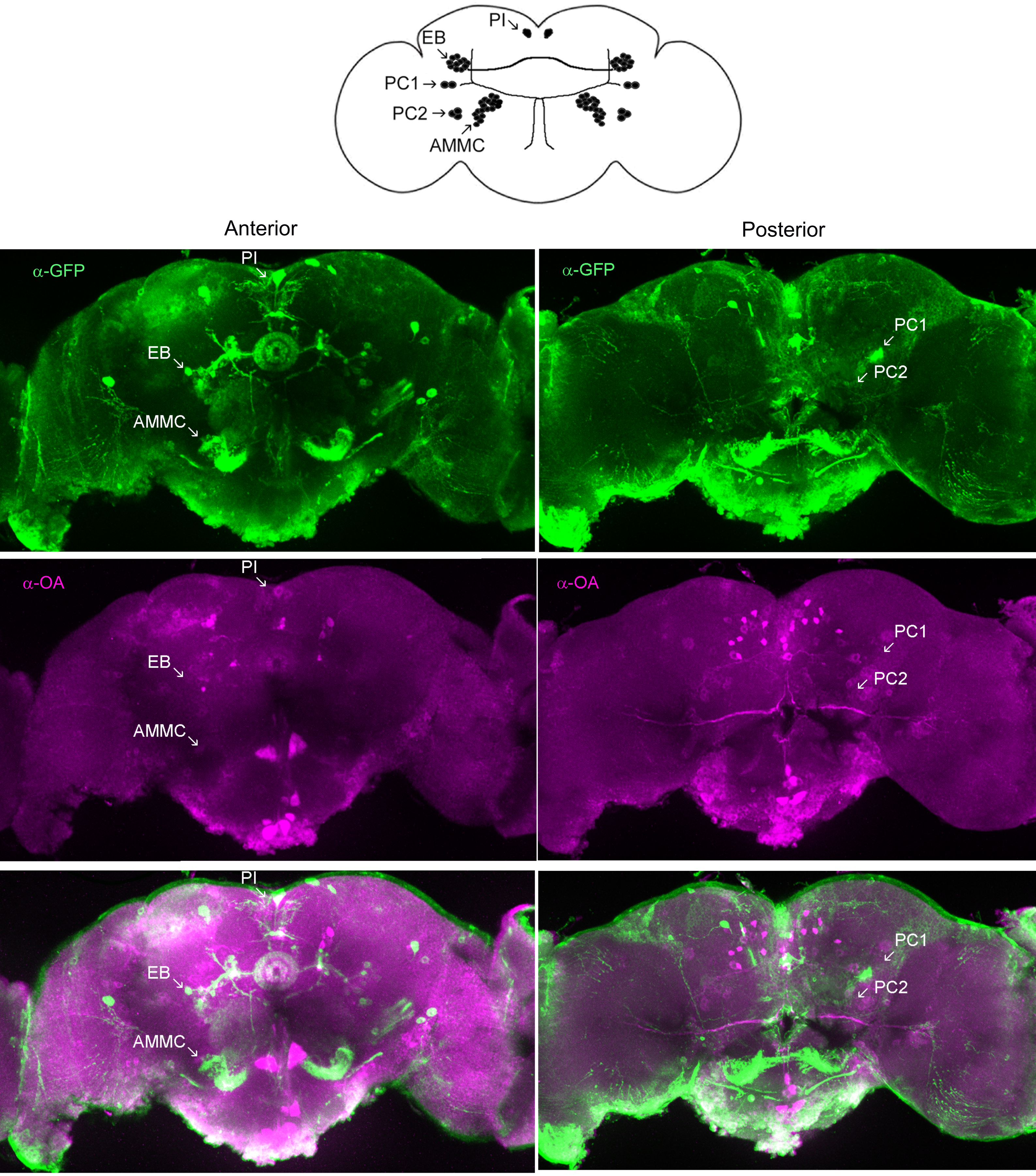

Figure S1: Comparison of 4.6-Tbh-Gal4 expression domain and octopamine expression
The Gal4 expression pattern of the 4.6-Tbh-Gal4 driver was visualized with a UAS-mCD8::GFP transgene (green) and compared to octopamine reactivity (magenta). Z-projection of the anterior and posterior part of the brain are shown. The antibody staining was conducted after (Busch et al., 2009).

Busch S, Selcho M, Ito K, Tanimoto H (2009) A map of octopaminergic neurons in the Drosophila brain. J Comp Neurol 513:643-667.
